# Effects of polymorphisms in *APOB*, *APOE*, *HSD11*β*1*, *PLIN4*, and *ADIPOQ* genes on lipid profile and anthropometric variables related to obesity in children and adolescents

**DOI:** 10.1590/1678-4685-GMB-2017-0195

**Published:** 2018-11-23

**Authors:** Caroline C. Gasparin, Neiva Leite, Luciane V. Tureck, Ricardo L.R. Souza, Gerusa E. Milano-Gai, Larissa R. Silva, Wendell A. Lopes, Lupe Furtado-Alle

**Affiliations:** ^1^ Laboratório de Polimorfismos e Ligação, Departamento de Genética, Universidade Federal do Paraná (UFPR) Curitiba, PR, Brazil; ^2^ Departamento de Educação Física, Universidade Federal do Paraná (UFPR) Curitiba, PR, Brazil

**Keywords:** *PLIN4* gene, *APOB* gene, *ADIPOQ* gene, *HSD11*β*1* gene, *APOE* gene

## Abstract

Genes can influence lipid profile and anthropometric variables related to obesity*.* The present study aimed to verify if variants of the *APOE*, *APOB*, *ADIPOQ*, *HSD11*β*1*, and *PLIN4* genes are associated with lipid levels or anthropometric variables in a sample comprised of 393 Euro-Brazilian children and adolescents. DNA was genotyped by TaqMan allelic discrimination assay. The ε4 and ε2 alleles of the *APOE* gene were associated respectively with lower high-density lipoprotein cholesterol (HDL-C) and low-density lipoprotein cholesterol (LDL-C) levels (*p*=0.015 and *p*=0.012, respectively), while the ε3 allele was associated with higher abdominal circumference (*p*=0.0416) and excess weight (*p*=0.0001). The G allele (rs846910) of the *HSD11*β*1* gene was also associated with excess weight (*p*=0.039). No other association was found. Our results indicate that the ε4 and ε2 alleles could contribute to lower HDL-C and LDL-C levels, respectively, furthermore, the ε3 allele and the G allele (rs846910) of *HSD11*β*1* gene may be risk factors for excess of weight.These findings are very important because we observed that some genetic variants influence the lipid profile and anthropometric variables early in life.

## Introduction

Dyslipidemia is closely related to the development of cardiovascular and cerebrovascular diseases, such as atherosclerosis, acute myocardial infarction, ischemic heart disease, and cerebrovascular accident, and therefore of great relevance for public health (ANVISA, 2011; Maria *et al.*, 2011). It is estimated that 53% of American adults have lipid abnormalities (Tóth *et al.*, 2012). In Brazil, according to Alcântara Neto *et al.* (2012), the prevalence of dyslipidemia among children and adolescents enrolled in the public school system was 25.5%. They also found a positive association between dyslipidemia and overweight (Alcântara Neto *et al.*, 2012). Worldwide, in 2015, the number of overweight children under five years old had been estimated at more than 42 million (WHO, 2016).

Dyslipidemias, as well as obesity, are mainly multifactorial traits, influenced by the environment, genetic factors, and life habits. Polymorphisms of the *APOB*, *APOE*, *ADIPOQ*, *PLIN4*, and *HSD11*β*1* genes are important examples of genetic causes associated with dyslipidemias and obesity. The genetic variants selected for this study seem to have functional effects, being involved in lipid metabolism and features related to obesity (Innerarity *et al.*, 1987; Soria *et al.*, 1989; Myant, 1993; Arita *et al.*, 1999; Foley, 2005; Greenow *et al.*, 2005; Heeren *et al.*, 2006; Lara-Castro *et al.*, 2007; Gambineri *et al.*, 2011; Richardson *et al.*, 2011).

The APOE glycoprotein plays an important role in metabolism, transport, and redistribution of molecules that carry cholesterol and other lipids (Poirier, 2005). It is encoded by a gene of the same name (19q13.2) and mediates the uptake of chylomicrons, very low-density lipoprotein (VLDL) and intermediate density lipoprotein (IDL) (Mahley, 1988; Weisgraber *et al.*, 1981). The ε2, ε3, and ε4 alleles (rs7412: NM_000041.3:c.526C > T and rs429358: NM_000041.3:c.388T > C) are combined in two important positions (Weisgraber *et al.*, 1981), producing therefore the three APOE major isoforms E2 (Cys 112, Cys 158), E3 (112 Cys, Arg 158), and E4 (Arg 112, Arg 158) (Foley 2005; Greenow *et al.*, 2005; Heeren *et al.*, 2006). ApoB-100 is encoded by the *APOB* gene (2p24.1) and it is present on the surface of LDLs (Blackhart *et al.*, 1986; Innerarity *et al.*, 1987). The R3500Q mutation (rs5742904: NM_000384.2:c.10580G > A) leads to diminished affinity for its receptor (Innerarity *et al.*, 1987; Soria *et al.*, 1989; Myant, 1993).

*PLIN4* (19p13.3; Ensembl 2015) participates in the Perilipin/ADRP/TIP47 (*PAT*) family of lipid storage droplet (LSD) proteins and appears to be involved in the storage of lipids in adipocytes (Brasaemle, 2007). The rs8887 (NM_001080400.1:c.*2270A > G) polymorphism is situated in the 3’UTR region of *PLIN4* gene. The less frequent allele of this site may induce a reduction of up to 20% in the PLIN4 level due to the creation of a miR-522 binding site in the 3’UTR region of the gene (Richardson *et al.*, 2011).

The human gene encoding adiponectin, *ADIPOQ* gene (3q27), is the most expressed gene in adipose tissue (Maeda *et al.*, 1996). Obesity, and in particular the accumulation of abdominal visceral fat, as well as type 2 diabetes mellitus, coronary disease, and arterial hypertension are accompanied by a reduction of serum adiponectin (Arita *et al.*, 1999; Lara-Castro *et al.*, 2007). The SNP of the *ADIPOQ* gene was rs1501299: NM_001177800.1:c.214+62G > T.

The *HDS11*β*1* gene (1q32.2; Ensembl, 2015) encodes the enzyme hydroxysteroid dehydrogenase type 1 (11β-HSD1), which is responsible for the conversion of inactive to active cortisol, in addition to regulating the interaction of cortisol with glucocorticoid receptors (Bujalska *et al.*, 1997). Transgenic rats that overexpress this enzyme in adipose tissue develop visceral obesity, insulin resistance, hyperglycemia, and hyperlipidemia (Masuzaki *et al.*, 2001). Among its polymorphisms are rs846910 (NM_001206741.1:c.-48-2986A > G), which corresponds to a non-coding region SNP of the *HSD11*β*1* gene, and rs12086634 (NM_001206741.1:c.332-29T > G), which occurs in an enhancer region in intron 3 (Gambineri *et al.*, 2011).

Hence, the aim of the present study was to investigate possible influences of the *PLIN4* (rs8887), *APOB* (rs5742904), *ADIPOQ (*rs1501299), *HSD11*β*1* (rs848910 and rs12086634), and *APOE* (rs7412 and rs429358; alleles ε2, ε3, and ε4) genes on lipid and glucose levels, abdominal circumference, and obesity in a sample of children and adolescents from a population in southern Brazil.

## Subjects and Methods

### Subjects

The sample was comprised of 393 Euro-Brazilians (13.54 ± 0.095 years old) living in Curitiba, PR, of which 143 were eutrophic and 250 overweight. Of these 393 individuals, 128 were girls (21.09% eutrophic and 78.91% overweight) and 265 were boys (43.94% eutrophic and 56.06% overweight). This study was approved by the Institutional Ethics Committee and informed consent was signed by participants and their parents or legal guardians.

Body mass index (BMI) was calculated as weight (kg) divided by the square of height (m). Age- and sex-specific BMI z-score and percentiles were calculated using CDC 2000 growth charts (Kuczmarski *et al.*, 2002). Eutrophic was defined as a < 85 percentile, overweight as a ≥85 percentile, and obesity as ≥95 percentile. The abdominal circumference (AC) was measured in centimeters (cm) at the level of the iliac crest. Thus, subjects were classified as eutrophic (percentile < 85) and overweight/obese (percentile ≥ 85) (Kuczmarski *et al.*, 2002).

Blood samples were collected in the morning after 12 hours of fasting to perform measurements of glucose (Glu), triglycerides (TG), total cholesterol (TC), and high density lipoprotein cholesterol (HDL-C) by standard automated methods. Low density lipoprotein cholesterol (LDL-C) levels were calculated using the Friedewald equation (Friedewald *et al.*, 1972), for TG levels below 200 mg/dL.

### Genotyping assays

DNA was extracted from peripheral blood by a salting-out method (Lahiri and Numberger, 1991) and was diluted to 20 ng/μL. All SNPs were genotyped by TaqMan allelic discrimination assay on StepOnePlus real time PCR systems (Applied Biosystems, USA). Each reaction contained 3.0 μL of Master Mix (2X), 1.7 μL of ultrapure water, 0.3 μL of primer and 3.0 μL of DNA. The reactions were performed according to the following protocol: 50 °C for 2 min, 95 °C for 10 min, and 50 cycles of 95 °C for 15 s and 62 ºC for 1 min.

### Statistical analysis

Samples were classified into two groups, eutrophic and overweight *(overweight + obese),* categorized into above and below the median for age, AC, Glu, TC, LDL-C, HDL-C and TG levels. Chi-square tests were performed using Clump (Jakobsson and Rosenberg, 2007) to test for Hardy-Weinberg equilibrium and to compare allele proportions between groups above and below the median and also between eutrophic and overweight. Logistic regression analyses were performed to identify variables influencing serum glucose, lipid concentrations, and AC. False discovery rate (FDR) corrections (Benjamini and Hochberg, 1995) were performed for multiple testing. The significance level adopted was 0.05 (5%).

## Results

A descriptive analysis of the sample, displaying the variables considered in this study, is shown in Table 1. Significantly higher frequencies were found for the ε4 allele in the group below the HDL-C median (*p*=0.0001), and for the ε2 allele in the group below the LDL-C median (*p*=0.0001). Furthermore the ε3 allele was associated with higher AC and excess weight (*p*=0.0001). The allele frequencies are shown in Table 2. Logistic regression analysis was done using stratified TC as below and above the median as the dependent variable, and for the polymorphisms analyzed (dominant model for *APOE* gene, in which ε4 is dominant over ε2; for the other polymorphisms, dominant, recessive, and additive models were tested), gender, AG, and anthropometric classification as independent variables. The same logistic regression analysis design was performed using LDL-C, HDL-C, TG, glucose, and AC as the dependent variable and maintaining the same independent variables. We identified the *APOE* gene ε4 allele as a contributing factor in reducing HDL-C levels (β = -0.29 ± 0.08, *p*=0.015) and the ε3 allele as a risk factor for higher AC measures (β = -0.24 ± 0.08, *p*=0.041). We also found that obesity and overweight are independent risk factors for higher triglyceride levels (β = 0.30 ± 0.08, *p*=0.021).

**Table 1:** Descriptive statistics for age, lipid profile, glucose, and abdominal circumference of the 393 individuals analyzed in this study.

Variable^*^	N^**^	Mean ± SE	Median	Variance	SD	Boys mean ± SE	Girls mean ± SE
Age	393	13.54 ± 0.095	13.96	3.56	1.89	13.54 ± 0.12	13.54 ± 0.17
HDL-C (mg/dL)	369	47.59 ± 0.89	46.00	116.203	10.78	45.44 ± 0.63	51.69 ± 1.02
LDL-C (mg/dL)	262	91.36 ± 1.85	87.50	898.187	29.97	89.93 ± 2.53	92.94 ± 2.72
TG (mg/dL)	367	99.17 ± 2.88	81.74	3055.92	55.28	96.06 ± 3.35	105.05 ± 5.41
TC (mg/dL)	262	162.67 ± 2.19	158.095	1261.96	35.52	160.42 ± 2.98	165.19 ± 3.24
Glu (mg/dL)	387	89.50 ± 0.56	89.00	120.842	10.99	90.60 ± 0.72	87.25±0.83
AC (cm)	291	83.69 ± 1.07	81.50	333.001	18.25	80.77 ± 1.21	92.25±1.94

* High Density Lipoprotein Cholesterol (HDL-C), Low Density Lipoprotein Cholesterol (LDL-C), Triglycerides (TG), Total Cholesterol (TC), Glucose (Glu), Abdominal Circumference (AC).

** 393 individuals were analyzed.

**Table 2:** Comparisons of allele frequencies between groups below and above the median for the analyzed variables, and between eutrophic and overweight/obese individuals.

Alleles	Above TC (mg/dL) median	Below TC (mg/dL) median	Above LDL-C (mg/dL) median	Below LDL-C (mg/dL) Median	Above TG (mg/dL) median	Below TG (mg/dL) median	Above HDL-C (mg/dL) median	Below HDL-C (mg/dL) median	Above AC (cm) median	Below AC (cm) median	Eutrophic	Overweight / Obese
ε2 *(APOE* gene)	3.57 ± 0.28 (6)	7.14 ± 0.55 (12)	1.83 ± 0.14 (3)	8.72 ± 0.66 (15) *p=*0.0001	4.84 ± 0.31 (12)	5.23 ± 0.32 (14)	4.92 ± 0.30 (13)	5.12 ± 0.32 (13)	6.25±0.49 (10)	2.31 ± 0.15 (5)	3.70±0.25 (8)	5.36 ± 0.29 (18)
ε3 *(APOE* gene)	82.14 ± 6.34(138)	81.55 ± 6.29 (137)	82.93 ± 6.48(136)	80.81 ± 6.16 (139)	78.22 ± 4.97 (194)	79.10 ± 4.83(212)	84.85 ± 5.22 (224)	72.44 ± 4.54(184)	71.88 ± 5.68(115) *p=*0.0001	71.30 ± 4.85(154)	76.39 ± 5.20 (165)	78.87 ± 4.30 (265) *p=*0.0001
ε4 *(APOE* gene)	14.29 ± 1.35 (24)	11.31 ± 1.31 (19)	15.24 ± 1.32 (25)	10.47 ± 1.30 (18)	16.94 ± 1.35 (42)	15.67 ± 1.24 (42)	22.44 ± 1.70 (27)	10.23 ± 0.88 (57) *p=*0.0001	21.87 ± 2.17 (35)	26.39 ± 1.95 (57)	19.91 ± 1.59 (43)	15.77 ± 1.12 (53)
G (rs846910 *HSD11*β*1* gene)	88.96 ± 2.52 (137)	90.85±2.25 (149)	88.19 ± 2.69 (127)	91.38 ± 2.13 (159)	88.75 ± 2.04 (213)	84.8 ± 2.27 (212)	87.5 ± 2.07 (224)	85.59 ± 2.28 (202)	87.86 ± 2.76 (123)	80.58±2.76 (166)	80.84 ± 2.69 (173)	89.42 ± 1.74 (279) *p=*0.039

* Only the significant findings (*p* < 0.05) are demonstrated in this table with *p*-value.

* High Density Lipoprotein Cholesterol (HDL-C), Low Density Lipoprotein Cholesterol (LDL-C), Triglycerides (TG), Total Cholesterol (TC) , Glucose (Glu), Abdominal Circumference (AC)

* The **n** of these groups are demonstrated between parentheses.

Furthermore, we observed that the A allele (rs846910) of the *HSD11*β*1* gene was associated with excessive weight (*p*=0.039, Chi-square test). It is known that there is variation in metabolic processes inherent to gender, so we conducted the same analyses separately for each gender. We observed that in girls the alleles ε2 and ε4 of the *APOE* gene were associated with LDL-C below the median (*p*=0.0001 by Chi-square test) and HDL-C below the median, independently of the other analyzed variables (β = -0.34 ± 0.08, *p*=0.0039) (Table 3). Furthermore, eutrophic girls had lower mean TG levels than obese or overweight girls (β = 0.30 ± 0.08, *p*=0.0039). Regarding boys, we observed that the ε2 allele is associated to lower LDL-C levels (*p*=0.019 by Chi-square test) (Table 3). 

**Table 3:** Comparisons of *APOE* allele frequencies between groups below and above the median for the analyzed variables, and between eutrophic and overweight/obese individuals stratified by sex.

Alleles in Girls Group	CT (mg/dl)		LDL-C (mg/dl)		TG (mg/dl)		HDL-C (mg/dl)		AC (cm)		Obesity status	
Above the median	Below the median	Above the median	Below the median	Above the median	Below the median	Above the median	Below the median	Above the median	Below the median	Eutrophic	Overweight/ obese
ε2 (APOE - rs7412 and rs429358)	2.33±0.25 (2)	7.14 ± 0.85 (5)	1.22 ± 0.14 (1)	8.11 ± 0.94 (6) *p*=0.0001	2.44 ± 0.27 (2)	6.25±0.70 (5)	5.21 ± 0.53 (5)	3.03 ± 0.37 (2)	0.00 ± 0.00 (0)	0.00 ± 0.00 (0)	3.12 ± 0.55 (1)	4.55 ± 0.40 (6)
E3 (APOE - rs7412 and rs429358)	83.72 ± 9.03 (72)	82.86 ± 9.90 (58)	84.15 ± 9.29 (69)	82.43±9.58 (61)	82.93 ± 9.16 (68)	81.25±9.08 (65)	87.5 ± 8.93 (84)	74.24 ± 9.14 (49)	66.67 ± 11.11 (24)	68.75 ± 12.15 (22)	84.38 ± 14.91 (27)	81.06 ± 7.06 (107) *p*=0.0001
ε4 (APOE - rs7412 and rs429358)	13.95 ± 1.74 (12)	10.00 ± 1.86 (7)	14.63 ± 1.75 (12)	9.46 ± 1.81 (7)	14.63 ± 1.86 (12)	12.5 ± 1.98 (10)	7.29 ± 1.16 (7)	22.73 ± 3.15 (15) *p*=0.0039	33.33 ± 5.56 (12)	31.25±5.52 (10)	12.5 ± 2.71 (4)	14.39 ± 1.60 (19)
Alleles in Boys Group	CT		LDL-C		TG		HDL-C		AC			
	Above the median	Below the median	Above the median	Below the median	Above the median	Below the median	Above the median	Below the median	Above the median	Below the median	Eutrophic	Overweight/ obese
ε2 (APOE - rs7412 and rs429358)	5.81 ± 0.63 (5)	6.38 ± 0.66 (6)	3.84 ± 0.44 (3)	7.84 ± 0.78 (8) *p*=0.019	6.79 ± 0.53 (11)	4.17 ± 0.30 (8)	5.00 ± 0.37 (9)	5.68 ± 0.43 (10)	7.35 ± 0.63 (10)	2.91 ± 0.22 (5)	3.80 ± 0.28 (7)	5.88 ± 0.41 (12)
ε3 (APOE - rs7412 and rs429358)	81.40 ± 8.78 (70)	79.79 ± 8.23 (75)	79.49 ± 9.00 (62)	81.37 ± 8.06 (83)	74.69 ± 5.87 (121)	79.16 ± 5.71 (152)	81.67 ± 6.09 (147)	72.73 ± 5.48 (128)	73.53 ± 6.30 (100)	71.51 ± 5.45 (123)	75.00 ± 5.53 (138)	77.45 ± 5.42 (158)
ε4 (APOE - rs7412 and rs429358)	12.79 ± 1.91 (11)	13.83 ± 1.98 (13)	16.67 ± 2.28 (13)	10.79 ± 1.67 (11)	18.52 ± 1.92 (30)	16.67 ± 1.47 (32)	13.33 ± 1.31 (24)	21.59 ± 2.01 (38)	19.12 ± 2.18 (26)	25.58±2.16 (44)	21.20 ± 1.82 (39)	16.67 ± 1.52 (34)

* Only the significant findings (*p* < 0.05) are demonstrated in this table with *p*-value.

* High Density Lipoprotein Cholesterol (HDL-C), Low Density Lipoprotein Cholesterol (LDL-C), Triglycerides (TG), Total Cholesterol (TC), Glucose (Glu), Abdominal Circumference (AC).

* The **n** of these groups are demonstrated between parentheses.

## Discussion

**Figure 1:**
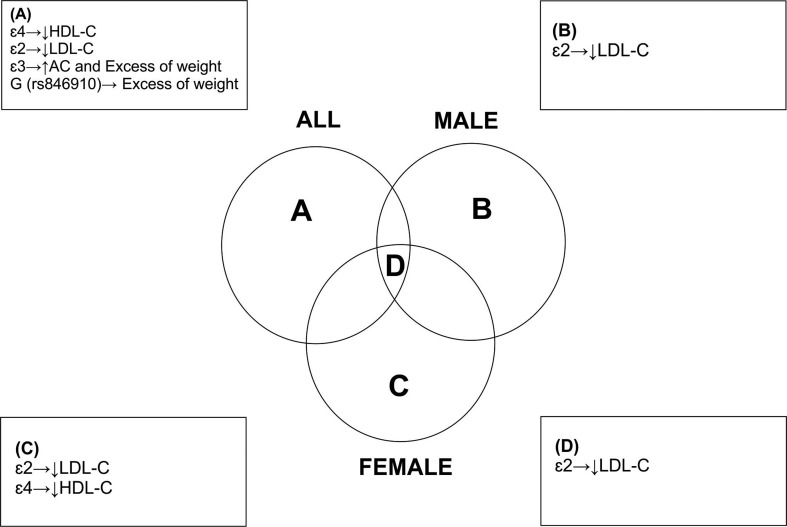
Relationships between allelic variants and analyzed variables.

Blood lipid levels are influenced by environmental and genetic factors (Crook, 2012), and it is known that LDL-C is the primary target for reducing cardiovascular risk (Catapano *et al.*, 2016). In our study, as shown in Figure 1, it was observed that the *APOE* ε2 allele was associated with lower LDL-C levels in the total sample, as well as in girls and boys, which is consistent with the known protective effect of this allele (Frikke-Schmidt *et al.*, 2000; Bennet *et al.*, 2007; Fuzikawa *et al.*, 2008; Ward *et al.*, 2009; Nascimento *et al.*, 2009; Bazzaz *et al.*, 2010; Ferreira *et al.*, 2010). Our finding is particularly relevant considering that the protective effect of the ε2 allele is usually observed in adults, but in our study we observed that it is also present in children and adolescents, and therefore can contribute to lower LDL-C levels early in life.

The *APOE*-ε4 allele seems to be associated with lower HDL-C levels, which support the notion that the ε4 allele is an atherogenic risk factor (Frikke-Schmidt *et al.*, 2000; Bennet *et al.*, 2007; Fuzikawa *et al.*, 2008; Ward *et al.*, 2009). Being related to lower HDL-C levels, responsible for cholesterol reverse transport, this allele could contribute to higher cholesterol levels, and this is especially worrisome in children, considering all possible and severe comorbidity (ANVISA, 2011; Maria *et al.*, 2011; Crook, 2012).

Besides its association with the lipid profile, some studies have demonstrated that the *APOE* gene influences characteristics of obesity (Volcik *et al.*, 2006; Tabatabaei-Malazy *et al.*, 2012). According to the Atherosclerosis Risk in Communities (ARIC) study, the apo E genotypes were associated with BMI following the order: apo E4 > apo E3 > apo E2 (Volcik *et al.*, 2006). Srinivasan *et al.* (1994), who analyzed a sample of children and adolescents, similar to this study, found that the apo E3 group showed significant associations with obesity measures and lipoprotein variables.

Our work is in agreement with Sun *et al.* (2016) who also found some increased variables, such as BMI and LDL-C, in ε3 allele carriers when compared to ε2 allele carriers in the non-metabolic syndrome group (Sun *et al.*, 2016). Some studies also have associated the ε4 allele with features related to obesity in different populations (Tabatabaei-Malazy *et al.*, 2012; Alharbi *et al.*, 2017). Therefore, these polymorphisms in the *APOE* gene influence both lipid profile and traits related to obesity. It is important to highlight the relevance of studies involving this gene, especially in the young. We found a relationship between the G allele of the *HSD11*β*1* gene rs846910 polymorphism and higher AC measurements. Some studies have demonstrated different effects of this polymorphism on serum lipid levels and other associated characteristics (Nair *et al.*, 2004; Duran-Gonzalez *et al.*, 2011; Dujic *et al.*, 2012). Different from this study, Durán-Gonzalez *et al.* (2011) observed an association between the A allele and higher triglyceride levels, and according to some studies this polymorphism could be associated to metabolic syndrome (Nair *et al.*, 2004; Duran-Gonzalez *et al.*, 2011; Dujic *et al.*, 2012). However, Turek *et al.* (2014) found that the A allele ws associated with higher HDL-C levels only in women. Furthermore, it is relevant to consider that a possible linkage disequilibrium might exist with another polymorphism in the *HSD11*β*1* gene, and another allele could be the cause of an altered lipid profile or features related to obesity (Malavasi *et al.*, 2010).

Although our study had relevant findings, we recognize that the small sample size is a limitation, thus generalizability should be done with caution, and studies with larger samples should be done. In summary, we found that in children and adolescents, as in adults, the ε4 and ε3 alleles could be considered a contributing factor for dyslipidemia and traits related to obesity, respectively, while the ε2 allele seems to be a protective factor, contributing to lower LDL-C and higher HDL-C levels. Furthermore, the *HSD11*β*1* gene G allele seems to be related to obesity. Considering that effects may start early in life, a precocious intervention could be planned, therefore preventing many complications resulting from altered lipid profile and obesity.

## References

[B1] Alcântara OD, Silva RCR, Assis AMO, Pinto EJ (2012). Fatores associados à dislipidemia em crianças e adolescentes de escolas públicas de Salvador, Bahia. Rev Bras Epidemiol.

[B2] Alharbi KK, Syed R, Alharbi FK, Khan IA (2017). Association of Apolipoprotein E polymorphism with impact on overweight university pupils. Genet Test Mol Biomarkers.

[B3] ANVISA (2011). Dislipidemia. Saúde Econ.

[B4] Arita Y, Kihara S, Ouchi N, Takahashi M, Maeda K, Miyagawa J, Hotta K, Shimomura I, Nakamura T, Miyaoka K (1999). Paradoxical decrease of an adipose-specific protein, adiponectin, in obesity. Biochem Biophys Res Commun.

[B5] Bazzaz JT, Nazari M, Nazem H, Amiri P, Fakhrzadeh H, Heshmat R, Abbaszadeh S, Amoli MM (2010). Apolipoprotein e gene polymorphism and total serum cholesterol level in Iranian population. J Postgrad Med.

[B6] Benjamini Y, Hochberg Y (1995). Controlling the false discovery rate: A pratical and powerful approach to multiple testing. J R Stat Soc.

[B7] Bennet AM, Di Angelantonio E, Ye Z, Wensley F, Dahlin A, Ahlbom A, Keavney B, Collins R, Wiman B, Faire U (2007). Association of apolipoprotein E genotypes with lipid levels and coronary risk. JAMA.

[B8] Blackhart BD, Ludwig EM, Pierotti VR, Caiati L, Onasch MA, Wallis SC, Powell L, Pease R, Knott TJ, Chu ML (1986). Structure of the human apolipoprotein B gene. J Biol Chem.

[B9] Brasaemle DL (2007). The perilipin family of structural lipid droplet proteins: Stabilization of lipid droplets and control of lipolysis. J Lipid Res.

[B10] Bujalska IJ, Kumar S, Stewart PM (1997). Does central obesity reflect “Cushing”s disease of the omentum?. Lancet.

[B11] Catapano AL, Graham I, De Backer G, Wiklund O, Chapman MJ, Drexel H, Hoes AW, Jennings CS, Landmesser U, Pedersen TR (2016). 2016 ESC/EAS guidelines for the management of dyslipidaemias. Eur Heart J.

[B12] Crook MA (2012). Plasma lipids and lipoproteins. In: Clinical Biochemistry and Metabolic Medicine.

[B13] Dujic T, Bego T, Mlinar B, Semiz S, Malenica M, Prnjavorac B, Ostanek B, Marc J, Causevic A (2012). Association between 11beta-hydroxysteroid dehydrogenase type 1 gene polymorphisms and metabolic syndrome in Bosnian population. Biochem Medica.

[B14] Duran-Gonzalez J, Ortiz I, Gonzales E, Ruiz N, Ortiz M, Gonzalez A, Sanchez EK, Curet E, Fisher-Hoch S, Rentfro A (2011). Association study of candidate gene polymorphisms and obesity in a young Mexican-American population from South Texas. Arch Med Res.

[B15] Ferreira CN, Carvalho MG, Fernandes APSM, Lima LM, Loures-Valle AA, Dantas J, Janka Z, Palotás A, Sousa MO (2010). Comparative study of apolipoprotein-E polymorphism and plasma lipid levels in dyslipidemic and asymptomatic subjects, and their implication in cardio/cerebro-vascular disorders. Neurochem Int.

[B16] Foley SM (2005). Update on risk factors for atherosclerosis: the role of inflammation and apolipoprotein E. Medsurg Nurs.

[B17] Friedewald WT, Levy RI, Fredrickson DS (1972). Estimation of the concentration of low-density lipoprotein cholesterol in plasma, without use of the preparative ultracentrifuge. Clin Chem.

[B18] Frikke-Schmidt R, Nordestgaard BG, Agerholm-Larsen B, Schnohr P, Tybjaerg-Hansen A (2000). Context-dependent and invariant associations between lipids, lipoproteins, and apolipoproteins and apolipoprotein E genotype. J Lipid Res.

[B19] Fuzikawa AK, Peixoto SV, Taufer M, Moriguchi EH, Lima-Costa MF (2008). Association of ApoE polymorphisms with prevalent hypertension in 1406 older adults: The Bambuí Health Aging Study (BHAS). Braz J Med Biol Res.

[B20] Gambineri A, Tomassoni F, Munarini A, Stimson RH, Mioni R, Pagotto U, Chapman KE, Andrew R, Mantovani V, Pasquali R (2011). A combination of polymorphisms in HSD11B1 associates with in vivo 11{beta}-HSD1 activity and metabolic syndrome in women with and without polycystic ovary syndrome. Eur J Endocrinol.

[B21] Greenow K, Pearce NJ, Ramji DP (2005). The key role of Apolipoprotein E in atherosclerosis. J Mol Med.

[B22] Heeren J, Beisiegel U, Grewal T (2006). Apolipoprotein E recycling: Implications for dyslipidemia and atherosclerosis. Arterioscler Thromb Vasc Biol.

[B23] Innerarity TL, Weisgraber KH, Arnold KS, Mahley RW, Krauss RM, Vega GL, Grundy SM (1987). Familial defective apolipoprotein B-100: Low density lipoproteins with abnormal receptor binding. Proc Natl Acad Sci USA.

[B24] Jakobsson M, Rosenberg NA (2007). CLUMPP: A cluster matching and permutation program for dealing with label switching and multimodality in analysis of population structure. Bioinformatics.

[B25] Kuczmarski RJ, Ogden CL, Guo SS, Grummer-Strawn LM, Flegal KM, Mei Z, Wei R, Curtin LR, Roche AF, Johnson CL (2002). 2000 CDC growth charts for the United States: Methods and development. Vital Health Stat.

[B26] Lahiri DK, Numberger JI (1991). A rapid non-enzymatic method for the preparation of HMW DNA from blood for RFLP studies. Nucleic Acids Res.

[B27] Lara-Castro C, Fu Y, Chung BH, Garvey WT (2007). Adiponectin and the metabolic syndrome: Mechanisms mediating risk for metabolic and cardiovascular disease. Curr Opin Lipidol.

[B28] Maeda K, Okubo K, Shimomura I, Funahashi T, Matsuzawa Y, Matsubara K (1996). cDNA cloning and expression of a novel adipose specific collagen-like factor, apM1 (AdiPose Most abundant Gene transcript 1). Biochem Biophys Res Commun.

[B29] Mahley RW (1988). Apolipoprotein E: Cholesterol transport protein with expanding role in cell biology. Science.

[B30] Malavasi ELV, Kelly V, Nath N, Gambineri A, Dakin RS, Pagotto U, Pasquali R, Walker BR, Chapman KE (2010). Functional effects of polymorphisms in the human gene encoding 11β-hydroxysteroid dehydrogenase type 1 (11β-HSD1): A sequence variant at the translation start of 11β-HSD1 alters enzyme levels. Endocrinology.

[B31] Maria V, Polachini BV, Zanardo-Giovana S, Ceni C (2011). Avaliação do perfil lipídico dos pacientes com dislipidemia atendidos no ambulatório de especialidades de nutrição da URICEPP. Perspectiva (Erechim).

[B32] Masuzaki H, Paterson J, Shinyama H, Morton NM, Mullins JJ, Seckl JR, Flier JS (2001). A transgenic model of visceral obesity and the metabolic syndrome. Science.

[B33] Myant NB (1993). Familial defective apolipoprotein B-100: A review, including some comparisons with familial hypercholesterolaemia. Atherosclerosis.

[B34] Nair S, Lee YH, Lindsay RS, Walker BR, Tataranni PA, Bogardus C, Baier LJ, Permana PA (2004). 11beta-Hydroxysteroid dehydrogenase Type 1: Genetic polymorphisms are associated with Type 2 diabetes in Pima Indians independently of obesity and expression in adipocyte and muscle. Diabetologia.

[B35] Nascimento H, Silva L, Lourenço P, Weinfurterová R, Castro E, Rego C, Ferreira H, Guerra A, Quintanilha A, Santos-Silva A (2009). Lipid profile in Portuguese obese children and adolescents: Interaction of Apolipoprotein E polymorphism with adiponectin levels. Arch Pediatr Adolesc Med.

[B36] Poirier J (2005). Apolipoprotein E, cholesterol transport and synthesis in sporadic Alzheimer’s disease. Neurobiol Aging.

[B37] Richardson K, Louie-Gao Q, Arnett DK, Parnell LD, Lai CQ, Davalos A, Fox CS, Demissie S, Cupples LA, Fernandez-Hernando C (2011). The plin4 variant rs8887 modulates obesity related phenotypes in humans through creation of a novel mir-522 seed site. PLoS One.

[B38] Soria LF, Ludwig EH, Clarke HRG, Vegat GL, Grundyt SM, Mccarthy BJ (1989). Association between a specific apolipoprotein B mutation and familial defective apolipoprotein B-100 (genetic disease/cholesterol metabolism). Genetics.

[B39] Srinivasan SR, Ehnholm C, Wattigney WA, Berenson GS (1994). Relationship between obesity and serum lipoproteins in children with different apolipoprotein E phenotypes: The Bogalusa Heart Study. Metabolism.

[B40] Sun Y, Wei R, Yan D, Xu F, Zhang X, Zhang B, Yimiti D, LI H, Sun H, Hu C (2016). Association between *APOE* polymorphism and metabolic syndrome in Uyghur ethnic men. BMJ Open.

[B41] Tabatabaei-Malazy O, Fakhrzadeh H, Qorbani M, Amiri P, Larijani B, Tavakkoly-Bazzaz J, Amoli MM (2012). Apolipoprotein E gene polymorphism and its effect on anthropometric measures in normoglycemic subjects and type 2 diabetes. J Diabetes Metab Disord.

[B42] Tóth PP, Potter D, Ming EE (2012). Prevalence of lipid abnormalities in the United States: The National Health and Nutrition Examination Survey 2003-2006. J Clin Lipidol.

[B43] Turek LV, Leite N, Souza RLR, Lima JK, Milano GE, Silva Timossi L, Osiecki ACV, Osiecki R, Alle LF (2014). Gender-dependent association of HSD11B1 single nucleotide polymorphisms with glucose and HDL-C levels. Genet Mol Biol.

[B44] Volcik KA, Barkley RA, Hutchinson RG, Mosley TH, Heiss G, Sharrett AR, Ballantyne CM, Boerwinkle E (2006). Apolipoprotein E polymorphisms predict Low Density Lipoprotein cholesterol levels and carotid artery wall thickness but not incident coronary heart disease in 12,491 ARIC study participants. Am J Epidemiol.

[B45] Ward H, Mitrou PN, Bowman R, Luben R, Wareham NJ, Khaw KT, Bingham S (2009). APOE genotype, lipids, and coronary heart disease risk: A prospective population study. Arch Intern Med.

[B46] Weisgraber KH, Rall SC, Mahley RW (1981). Human E apoprotein heterogeneity. Cysteine-arginine interchanges in the amino acid sequence of the apo-E isoforms. J Biol Chem.

[B47] World Health Organization - WHO (2016). Physical activity recommendations.

